# Artificial Illumination

**Published:** 1887-03

**Authors:** 


### EDITORIAL.

ARTIFICIAL ILLUMINATION.

The matter of artificial illumination is one that concerns man's well-being in many ways, and deserves much thought.

It is not the purpose of this article to deal with the question of relative expense and consequent economy, important as that is, but rather to consider the influence upon man's well-being, both in the general and in the local effects produced by different methods of illuminating.

So far as the general influence on the human body is concerned there are certain important requisites. These will vary somewhat with the attendant circumstances under which the light is to be used. The same conditions may not be equally required in the study of the professional man, the counting-room of the merchant, and the factory where artisans are employed. All of them do require proper amount, and right direction of the light, and steadiness of it. These are important desiderata so far as the eyes are concerned.

As influencing the general health, the consumption of the oxygen of the air and the substitution for it of noxious gases, especially when ventilation is defective, are matters of no small import.

The most generally used methods of illumination may probably be classed as, first, by oil, animal or vegetable; second by gas, and third by electricity, these having led to the almost total disuse of other modes of illuminating. Petroleum when purified is comparatively free from danger; furnishes good illumination, and a steady flame. Its consumption of oxygen and giving off of noxious gases and heat are its chief objections. Next in order comes gas as usually furnished in centers of population. The advantages and disadvantages of this differ very much, owing to various circumstances, as the material from which it is manufactured, defects in methods of distribution, etc. The good illumination from it generally and the convenience attending its use have led to its extensive use for illuminating purposes. The objections to it, viewed exclusively from the view of its influence on man's physical condition, are general and local. When there is escape of gas into man's dwelling-place, as from defective pipes or otherwise, the deleterious effect is shown in various ways upon the human system, even resulting in death when breathed in a moderately diluted state. When more diluted with air, the deleterious influence of breathing it varies only in degree. It consumes, also, oxygen from the air, and gives off poisonous gases and heat and unconsumed carbon, thus vitiating the air breathed, impoverishing the blood and depressing the system.

As usually employed, it burns with more or less unsteadiness of flame, which is very trying to the eyes. The amount and the direction of this illumination are often very objectionable.

The third method, by electricity, which is now coming into more general use, appears to obviate many of the objections which attend the use of most of the other methods of illumination.

The two forms in which it is most generally used are what are called the *arc light* and the *incandescent light.* Both forms possess the advantages of not consuming the oxygen of the air and substituting therefor deleterious gases, or permitting unconsumed carbon to escape, and they do not greatly heat confined rooms, whilst giving good illumination. The arc light possesses advantages over the incandescent light in having greater illuminating power, in consequence of which it seems to be better adapted to illuminating large spaces; but it generally possesses the disadvantage of being more or less flickering, and therefore is trying to the eyes. Because of its brilliancy it should be placed as high as practicable, especially in public thoroughfares. The paleness of the light given by the arc light makes it in some respects a less agreeable light. The incandescent light is slightly tinted, and therefore is more agreeable. It is more steady, gives a less dazzling light, and seems, in many respects, better suited for the illumination of smaller spaces.

Dr. J. A. Andrews, of New York, made a carefully prepared report, last year, to the *American Ophthalmological Society*, on the electric light, from which the following practical points are extracted. He says:

It is of practical importance to learn something about the relative effect of the different forms of artificial light on the human retina. All forms of artificial light contain, as compared with daylight, an excess of waves of long wave-length,—i. e. they are of a *yellowish* hue. In the electric light the short-wave rays predominate,—i. e. the violet rays.

As far as mere *color* is concerned, the *electric light* approaches nearer to that of the *sun* than does the gas or lamp flame. But which has the more to do in producing evil effects on the eyes after long and continuous work by artificial light—*color or heat?*

Gas emits the greatest amount of heat; petroleum lies between gas and electric light.

Tyndall has shown that the value of the luminous radiation from the flame of oil, gas, and the electric light is as follows:

| | Luminous. | Obscure. |
|---|---|---|
| Oil flame | 3 | 97 |
| Gas flame | 4 | 96 |
| Electric light | 10 | 90 |

We have therefore, with the *electric light*, the *maximum of light* with the *minimum of heat*.

Chardonnet (Vision des radiations ultra-violettes, Compt. Rend. hebd. des Seances de l'Acad., No. 8) has recently made some interesting investigations on the absorption of ultra-violet rays by the media of the eye, particularily by the crystalline lens, this absorption not being done without fatiguing the eye, especially when it concerns the long and brilliant spectrum of the *electric arc*. But he found on photographing the spectrum of the incandescent electric light, that the spectrum hardly passed beyond the visible spectrum; hence he inferred that this incandescent electric light saved the media before the retina the labor of absorbing and diffusing the ultra-violet spectrum. I do not believe that we should attach much practical importance to this explanation. It is easy to convince one's self that the inconstant and unsteady intense glare of the *arc electric light*, only faintly modified by an *opal* or *ground-glass globe*, is extremely distressing to the eyes.

So far all the cases of injury to the eye from the electric light recorded in medical literature have resulted from exposure of the eye in close proximity to the *arc electric light*, and this has, in every instance, been of a very intense brilliancy, generally that used for street-illuminating purposes.

So far as I have been able to ascertain, no case of accident to the eye has been reported as having been caused by the *incandescent electric light.* Of the 1100 persons whom I have examined and observed during the past year who work by day or by night for many hours by the *incandescent electric light* of *Edison's lamp*, there was not a single instance of injury to the eye; but, on the contrary, I was surprised to find that a considerable number of these persons, with a high degree of myopia and choroidal changes, were absolutely comfortable while working by this light, and they expressed themselves as having experienced a great improvement in the condition in their eyes since they had begun to work by the electric light (Edison lamp of twelve to sixteen-candle power), always shaded from the eyes, the light being thrown down upon the work from a short elevation. Many persons among type-setters and copyists have told me that formerly, when gas was in use in the establishment in which they worked, they went home from their work with their eyes red, watering, and aching, and this was especially the case among short-sighted persons, but that they could work longer by the Edison light (the arc light distressing them), and without discomfort. Of course, every form of artificial light is more or less dazzling when the source of light casts its image on the retina. We certainly can avoid this dazzling in the gas or petroleum flame by means of an appropriate shade; but, in order to secure the requisite amount of illumination, we must either approach the light close to the head, and thereby expose the eyes to the undesirable radiant heat, or increase the illumination at a greater distance, and thereby make an increased demand upon the vital parts of the atmospheric air in the room, thus vitiating the latter, which is a very undesirable equivalent. The amount of heat radiated by the electric light is very unimportant; and when, after sunlight, we add to its unrivalled qualities as an illuminator the steadiness and absence of combustion of the *incandescent lamp of Edison*—there being, therefore, no consumption or vitiation of the atmospheric air induced by it—together with the practical clinical fact that it is a greater comfort for even persons with existing eye affections to work by this light than by any other artificial illuminator, we must, from a therapeutic point of view, concede to the incandescent electric light advantages not possessed by any other artificial means of illuminating; and any one who has given the subject thoughtful consideration will at once see the great blessing which would come from introducing this kind of light into our *night-schools*, *theatres*, *public halls*, and those innumerable *counting-rooms* in great cities where large numbers of human beings work by artificial light during the entire day or night, the demand upon the atmospheric air by the individuals alone being, in the absence of adequate means of ventilation, and in the majority of instances with very little ventilation at all, in these overcrowded places, terrible enough without the additional mischievous agent in vitiating the atmospheric air present in the many *gas flames.* The advantage of using the incandescent light in our ophthalmoscopic dark-chamber must be evident to every ophthalmic surgeon.

My conclusions are as follows:

The *arc electric light* is not so objectionable on account of its intense brilliancy, which can be modified by means of an opal or ground-glass shade or globe.

The gazing at the *arc lamp* for many seconds is attended with great risk to the eyes. The *arc light*, in its present state, should be positively rejected as unsuitable and actually harmful to the human eye, particularly on account of its unsteadiness.

The *incandescent light of Edison*, because of its steadiness, adequate power, and composition, is safe, and occupies at present the first position as a means of artificial illumination. By its use the accommodation of the eye is less taxed than by other illuminators.

The light may advantageously be placed on the table in *front of* the person using it, but it should *never shine directly into the eye*, but always be *completely hidden* from the eyes by means of an opaque shade made of glass or tin, and the light be thrown down upon the work. For obvious reasons, it is further believed that the incandescent electric light is especially beneficial to myopes.

Discussing the report, DR. AGNEW said: I would ask for a moment to say that Columbia College, of which I am a trustee, has built a very large room in connection with its library for the night use of students. I had something to do with the introduction of the incandescent light into it. Each table is provided with an electric light, over which is placed a conical shade to protect the eyes from the direct light. For two winters we have kept the library room open until ten o'clock every night except Sunday night, and our students have been encouraged to come there. The library was also thrown open to the public, a simple introduction with a card being all that was necessary. Persons engaged in literary pursuits have come in considerable number to the library to study. A number of persons with sensitive eyes have come there to test the effect of the incandescent light, and have obtained permission to do continuous work there every evening. They found that they could work longer with less detriment to the eyes there than in any other place to which they had access. They have come from rooms where they have had gas and kerosene lamps, many of them having previously secured what they had supposed to be the best means of illumination. The introduction of the incandescent electric light there has proven of great advantage. I agree with the writer in all that he has said bearing upon that particular matter. This matter is worthy of the consideration of those having to do with colleges or schools requiring work at night. Aside from the fact of its not vitiating the air, the steadiness of the ight, and the fact that it is directed upon the work and shielded from the eye commends it. I think that air-poisoning has a good deal to do with the progress of eye diseases.

